# An Examination of Racial Bias in Scoring the Autism Diagnostic Observation Schedule (ADOS) Module 3: An Item Response Theory Analysis

**DOI:** 10.1002/aur.70155

**Published:** 2025-12-23

**Authors:** Yuen Yvonne Yu, Austin Wyman, Calliana J. Faulk, Lizzy J. Fulop, Rebecca L. Greenberg, Rachel M. Benecke, Lauren K. Steinbeck, Jessica Foy, Caitlyn Kim, George O. Emory, Eric A. Storch, Casey J. Zampella, Benjamin E. Yerys, Robert T. Schultz, Julia Parish‐Morris, John D. Herrington, Caitlin C. Clements

**Affiliations:** ^1^ Graduate School of Applied and Professional Psychology, Rutgers The State University of New Jersey Piscataway New Jersey USA; ^2^ Center for Autism Research Children's Hospital of Philadelphia Philadelphia Pennsylvania USA; ^3^ Department of Psychology University of Notre Dame South Bend Indiana USA; ^4^ Department of Psychiatry and Behavioral Sciences Baylor College of Medicine Houston Texas USA; ^5^ College of Medicine University of Florida Gainsville Florida USA; ^6^ College of Public Health and Health Professions University of Florida Gainsville Florida USA; ^7^ Department of Child and Adolescent Psychiatry and Behavioral Sciences Children's Hospital of Philadelphia Philadelphia Pennsylvania USA; ^8^ Department of Psychological Sciences University of Connecticut Storrs Connecticut USA; ^9^ Advancing Transition and Learning for Adult Success Center Children's Hospital of Philadelphia Philadelphia Pennsylvania USA; ^10^ Department of Psychiatry Perelman School of Medicine at the University of Pennsylvania Philadelphia Pennsylvania USA

**Keywords:** ADOS, autism, differential item functioning, item response theory, racial bias

## Abstract

Given the rising prevalence of autism among racial minority children in the United States, but persistent service use disparities, this study examines potential bias in specific items from the autism diagnostic observation schedule (ADOS), a highly regarded autism evaluation. We leveraged unidimensional item response theory graded response models and a sample of 735 children to analyze the differential item functioning (DIF) of items within ADOS Module 3. Three items showed significant signs of racial bias: A1 (overall language level), A5 (offers information), and D5 (compulsions and rituals). On these items, Black/African American and Asian children were usually more likely to be rated as showing autistic behaviors than White children with similar autism levels. The impact of racial bias on the item score was small, and the impact on the overall test score was even smaller: on a scale of 0–48 points, the effect of racial bias was estimated at 0.23 total points for Black/African American children and 0.16 points for Asian children. Furthermore, none of the items showing significant bias contribute to the autism classification algorithm. This analysis suggests a small but detectable amount of bias in several specific ADOS items, but not in items central to informing an autism diagnosis. Thus, bias appears statistically, but not clinically, significant. This contributes to examinations of racial bias in the ADOS as the first analysis of Asian children and the first in‐depth look at all items in the most commonly used version among school‐aged children.

## Lay Summary

In the ADOS, a widely used autism assessment, school‐age children with fluent language who are Asian or Black/African American are slightly more likely to be rated as showing autistic behaviors on 3 out of 24 items. These small differences appear on items that do not affect the total score. Still, clinicians should be aware of possible bias when evaluating Asian and Black/African American children, especially when scoring ADOS Module 3 items A1 (overall language level), A5 (offers information), and D5 (compulsions and rituals).

## 1 Introduction

For the first time in the United States, a higher prevalence of Autism Spectrum Disorder (autism) was reported for Black/African American and Asian children than White children in 2020 (Maenner 2023), and this pattern continued in following years (Shaw et al. 2025). Improvements in awareness, screening, and stigma likely contributed to the shift (Harris 2023; Kilbourne et al. 2006; Weitlauf et al. 2024). Black/African American and Asian autistic children still face significant racial disparities in many aspects of health care in the United States, and Asian children in particular have received little attention (Smith et al. 2020).

While the median age of autism diagnosis in the United States is 4 years, 1 month (Maenner 2023), Black/African American children receive a diagnosis at approximately 5 years, 4 months. This gap means that Black/African American children must wait an additional 27 months on average to initiate autism‐focused early intervention and do not receive a diagnosis until nearly 42 months after first parental concern (Constantino et al. 2020). Black/African American autistic children have less access to services than White children (Liu et al. 2023) and receive lower quality of care (Smith et al. 2020). Asian autistic children have received less attention in health disparity research, and research suggests that they are less likely to receive occupational or speech‐language therapies than White children (Smith et al. 2020; Irvin et al. 2012). Given these persistent race‐linked autism service disparities and the evolving landscape of autism diagnosis in the United States, there is an urgent need to identify and understand mechanisms underlying autism diagnostic disparities (Kilbourne et al. 2006). Examining potential bias in autism diagnostic tools is a place to start.

### 1.1 Autism Diagnostic Observation Schedule (ADOS)

The Autism Diagnostic Observation Schedule (ADOS) is widely regarded as a “gold standard” source of information about a child's behavior and social skills during the autism diagnostic process (Lord et al. 1999; Lord et al. 2012). However, the play‐based, semi‐structured ADOS is scored by a clinician and thus may be susceptible to bias. The ADOS version appropriate for verbally fluent children from 4 to 15 years old, ADOS Module 3, consists of activities tailored to the child's age and language level (e.g., make‐believe play with action figures, conversations about emotions and bullying) that are designed to elicit certain behaviors relevant to autism (e.g., intonation of speech, integration of facial expression with eye contact, quality of rapport, turn‐taking during conversation). After the 45–60 min long social interaction, the clinician rates the “abnormality”^1^ of the specified behaviors (Lord et al. 2012). Each score includes a detailed description with examples to guide clinician ratings. Training is rigorous and includes a 2‐ to 4‐day training, followed by review and feedback on multiple videotaped administrations and scores. A “reliable” administrator has achieved consistent administration fidelity and 80% scoring reliability (Carr 2013). A subset of ADOS item scores is summed to create the total score (“algorithm score”), which has cut‐offs for classification of autism, autism spectrum, and non‐spectrum (Lord et al. 2012). ADOS classification does not constitute a diagnosis, but the clinician integrates this information with multiple other sources such as a developmental history, caregiver interview, and cognitive testing (Bishop and Lord 2023).

### 1.2 Review of Bias in ADOS Scoring

In a fair, unbiased test, the proportions of children receiving any given ADOS item score would be equal across races, assuming equivalent latent autism trait levels across groups (e.g., if 35% of White children receive a score of 1, it is expected that 35% of Black/African American and 35% of Asian children receive a score of 1 on that item). The expectation of equal proportions might require adjustment if the subgroups have different latent levels of autism. Item Response Theory (IRT) and Differential Item Functioning (DIF) account for the child's underlying level of autistic traits (“latent trait level” calculated using all ADOS item scores) when computing the expected proportions of scores for each racial group. Proportions that diverge from the expected proportions after controlling for underlying autism levels indicate bias (i.e., DIF).

Two previous studies identified racial bias in ADOS item scores, in opposite directions. Both used IRT and DIF analyses to investigate whether racial subgroups vary in their probabilities of having certain ADOS items endorsed after controlling for their latent construct levels. Harrison and colleagues (Harrison et al. 2017) used the large Simons Simplex Collection sample (*n* = 2458) to explore bias between Black/African American and White participants in the original commercial version of the ADOS (ADOS), administered by research‐reliable clinicians, in the 10 items that are common across all modules. They found two factors that mapped onto the social communication domain and restricted or repetitive interests and behaviors domain (RRBI), and three items with statistically significant bias: unusual eye contact, stereotyped/idiosyncratic use of words or phrases, and immediate echolalia. The impact of the DIF was determined to be meaningful for the RRBI domain only; Black/African American children showed increased RRBI trait levels with a small to medium effect size, which disappeared after controlling for DIF (Harrison et al. 2017). Kalb and colleagues (Kalb et al. 2022) reported on potential Black‐White racial item bias in a clinical setting, where the newer ADOS‐2 was administered to 6269 patients by clinicians who were not necessarily research‐reliable administrators (as is common in clinical settings). They examined 14 items from Modules 1–3. They found one factor (i.e., a unidimensional model) fit the data best. Eight items showed statistically significant bias, including several from Module 3: facial expressions, quality of overtures, and stereotyped language. Stereotyped language was the only item to overlap with results from Harrison et al., but in contrast, scores of Black patients underestimated instead of overestimated their latent trait levels. In the analysis by Kalb et al., the size of the DIF was determined to be small, and the impact on the ADOS‐2 total score was less than one point (Kalb et al. 2022; Williams 2022).

### 1.3 Objectives

Both previous reports focused on the subset of items used in the ADOS scoring algorithm across modules (versions) of the ADOS administered to ages 18 months and above. Items outside the algorithm do not contribute to the total score but do contribute to the overall clinical impression. Clinicians frequently reference these items when forming diagnostic impressions, completing the DSM‐5‐TR checklist, and writing clinical reports. This paper will explore DIF in 24 items in Module 3, the version commonly used with school‐aged verbally fluent children. This paper also offers the first analysis of potential bias affecting Asian children. Thus, the paper aims to detect and quantify differential functioning in ADOS Module 3 items across Asian, Black/African American, and White children.

## 2 Method

### 2.1 Participants

Data were analyzed from 14 research studies conducted at the Center for Autism Research at the Children's Hospital of Philadelphia from 2009 to 2024 (IRB # 23–021354). Participants provided informed consent and were included in this secondary analysis if they identified as White, Black/African American, or Asian, and completed the ADOS or ADOS‐2 Module 3 as this module provided the largest sample size (*n* = 735, ages 5–19 years). No other racial groups in the dataset possessed sufficient data for analysis. Participants were classified as autistic if they met the Diagnostic and Statistical Manual of Mental Disorders, Fifth Edition (DSM‐5; APA, 2013) criteria for Autistic Spectrum Disorder, confirmed in‐house by an expert clinician integrating multiple sources of information. All ADOS administrations were conducted or directly supervised by a licensed clinical psychologist with significant expertise in autism diagnostics and ADOS administration. Consistent with previous analyses that combined different versions of items across modules (Harrison et al. 2017; Kalb et al. 2022), we combined ADOS and ADOS‐2 (henceforth, “ADOS”) items for the primary analysis to maximize power, and all ADOS administrations were rescored with the current scoring algorithm following standard procedures (Hus et al. 2014).

### 2.2 Measures

#### 2.2.1 Autism Diagnostic Observation Schedule (ADOS) Module 3

The ADOS is a gold‐standard, clinician‐administered assessment widely used in clinical and research settings as part of an autism diagnostic assessment. Module 3 is typically administered to verbally fluent children aged 4–15 years. The original edition of the ADOS (Lord et al. 1999) was updated to the ADOS‐2 in 2012 (Lord et al. 2012); minor edits were made to item text and administration procedures, and one item was added (B8, Amount of Social Overtures/Maintenance of Attention). Module 3 includes 29 items that assess five domains of behavior: (A) language and communication; (B) reciprocal social interaction; (C) imagination; (D) stereotyped behaviors and restricted interests; and (E) anxiety, tantrums, and activity level. See supplement for additional description of ADOS procedures, items, and scoring.

#### 2.2.2 Additional Measures

As a standard part of research participation across studies, participants also completed a general intelligence test (Differential Abilities Scale—2nd Edition, Wechsler Abbreviated Scales of Intelligence—2nd Edition, or Wechsler Intelligence Scale for Children—4th Edition), (Elliott 2007; Wechsler 2003; Wechsler 2011) and two questionnaires. The Social Communication Questionnaire—Lifetime (SCQ) is a 40‐item caregiver‐report autism screening questionnaire that focuses on the child's developmental history and evaluates their social functioning and communication skills. Scores of 12 and greater are considered indicative of a potential autism diagnosis (Rutter et al. 2003). The Social Responsiveness Scale, Second Edition (SRS‐2) School‐Age is a 65‐item caregiver‐report questionnaire that assesses autism‐related social ability in individuals aged 4 to 18 years. (Constantino and Gruber 2012).

### 2.3 Analysis

#### 2.3.1 Item Selection and Scoring

ADOS and ADOS‐2 Module 3 items (j = 24) were analyzed from the domains of (A) Language and Communication, (B) Reciprocal Social Interaction, and (D) Stereotyped Behaviors and Restricted Interests, as these domains are most relevant to the autism diagnostic criteria. One item (B8 Amount of Social Overtures/Maintenance of Attention) was not analyzed because it did not appear in the original edition of the ADOS. ADOS scoring conventions were followed (i.e., converting scores of 3 to 2, and converting scores of 7, 8, or 9 to 0). One item, B1 Eye Contact, was scored dichotomously as 0 or 2, per convention (i.e., clinicians never assign a score of 1). All other item scores were polytomous and scored as 0, 1, or 2; clinicians are trained to score a 0 if the rated behavior “shows no evidence of abnormality,” and to score a 2 if the rated behavior “is definitely abnormal.” (Lord et al. 2012).

#### 2.3.2 Model Selection

Confirmatory 1‐ and 3‐factor graded response models were fit to the 24 items from the domains of Language and Communication, Reciprocal Social Interaction, and Stereotyped Behaviors and Restricted Interests. Two separate 3‐factor models were fit: one allowed factors to correlate freely, and the other did not (i.e., not allowing covariance). The resulting three models were compared on the following goodness‐of‐fit indices: second‐order marginal statistic (M2, lower values represent a better fit); the root mean square error of approximation (RMSEA, values closer to 0 represent a better fit); the Tucker–Lewis index (TLI, values closer to 1 represent a better fit); and the comparative fit index (CFI, values closer to 1 represent a better fit). Analyses were implemented using the mirt package in R (Chalmers 2012).

#### 2.3.3 Differential Item Functioning Analysis

A multiple‐group model was employed to compare Asian and Black/African American groups to the White (reference) group. An Auxiliary Test, or All Other as Anchor method (Woods 2009) based on the likelihood‐ratio test, was implemented in order to detect and analyze DIF. First, items were fitted into a baseline model where item parameters including latent means and variances across groups were explicitly defined to be constrained. Items were inspected by allowing parameters of each item to be freely estimated one at a time to detect DIF with a slightly less restrictive model, where DIF would be flagged when *p* < 0.05. For the final model, non‐DIF items from the baseline model were explicitly specified as anchor items, which were used as references at the latent construct level to capture DIF effects by constraining all freely estimated parameters. The All Other as Anchor method is appropriate for the present study due to its low Type I and Type II errors under exploratory research conditions, as true anchor items are unknown (Wang and Woods 2017). Parameters estimated for each item included: discrimination (*a*), the ability of the item to discriminate between test‐takers of different latent trait levels; the difficulty of a 1 score (*b*_1_), the mean latent trait level (standardized) of test‐takers who scored a 1; and similarly, the difficulty of a 2 score (*b*_2_). Analyses were implemented using the mirt package in R (Chalmers 2012).

##### 2.3.3.1 Item‐Level Effect Size Calculation and Interpretation

We employed the effect sizes of the signed item difference (SIDS), unsigned item difference (UIDS), and expected standardized score difference (ESSD) to determine the magnitude of DIF at the item level. The SIDS effect size represents the mean difference between the focal and reference group for the specified item, averaged across participants. SIDS allows cancellation of opposing effects if some participants show positive effect sizes while others show negative effect sizes (Meade 2010). In contrast, the UIDS does not allow cancellation across participants. The UIDS reflects the mean absolute difference between the focal and reference groups for the specified item (Meade 2010). Both SIDS and UIDS are reported in units of the item score ranging from 0 to 2. The ESSD expresses SIDS in standard deviation units and thus can be interpreted as an effect size statistic analogous to Cohen's *d* (Meade 2010). Analyses were implemented using an open‐source mirt wrapper (Williams 2023).

##### 2.3.3.2 Test‐Level Effect Size Calculation and Interpretation

We quantified the impact of DIF at the test level using the Unsigned Expected Test Score Differences in the Sample (UETSDS)—the mean absolute difference in total ADOS score after allowing for cancellation within a participant (Meade 2010). In other words, if one DIF item resulted in a two‐point decrease in total ADOS score while another DIF item resulted in a two‐point increase in total ADOS score, the participant's UETSDS would be zero. UETSDS is expressed in ADOS point units. Of note, the total ADOS score in this analysis reflects the total of all items (24 items, total score range 0–48), but only a subset of items (“algorithm items”) are included in the traditional ADOS total score. While it would be possible to account for these two scenarios, it was not necessary given our pattern of results. Standard errors were computed for UETSDS scores using 1000 bootstrap samples, which allows for a more robust estimation of error and the computation of confidence intervals for future replication studies (Lai 2021).

##### 2.3.3.3 Uniform and Nonuniform DIF

DIF can present as uniform or nonuniform in an item. In uniform DIF, one group has higher scores than the other group, equally across all latent levels of autism. In nonuniform DIF, the size and possibly the direction of the DIF depend on the latent level of autism. For example, Asian children with low levels of autism could score lower on a particular item than White children with equivalent latent levels of autism, but on the same item, Asian children with high levels of autism score higher than White children with equivalent levels. When DIF is uniform, the absolute values of UIDS and SIDS are equal. When UIDS is larger than the absolute value of SIDS, the DIF is nonuniform, and cancellation occurs across respondents' DIF; the differential relationship is conditioned upon latent trait levels.

#### 2.3.4 Power Analysis

A post hoc power analysis was conducted using Monte Carlo simulation. First, we generated modeled population data that contained known DIF effects. Next, from the population dataset, we sampled 1000 data sets that matched our empirical dataset on sample size, group size, number of anchor items, and other parameters. Then, the analysis was conducted on each sampled dataset. Overall power was determined by the minimum individual power for each item, which was determined by the number of times the item was significant (likelihood ratio test) out of the total number of repetitions. One thousand repetitions were selected for sufficient parameter stability.

## 3 Results

### 3.1 Sample Characteristics

Participants included 625 White, 89 Black/African American, and 21 Asian children before exclusions (Table 1). Participants were excluded from the final analysis if they scored a 2 on item A1 Overall language level, indicating they did not show enough language complexity during the ADOS for Module 3 to be the appropriate module. The data set admittedly suffers from the overrepresentation of White males, as unfortunately is the case in most available large ADOS datasets at this time (e.g., Simons Simplex Collection 3% Black/African American and 3% Asian; National Database for Autism Research 11% Black/African American and 4% Asian). While modern and more diverse large ADOS data sets are being collected, the present data set can address the urgent need to tackle health disparities and also generate hypotheses for future bias analyses with awaited diverse ADOS datasets.

**TABLE 1:** Participant characteristics.

	Asian	Black/African American	White
*N*	21	87	618
Male	13	65	475
Female	8	24	150
Age (Years)	11.6 (2.3)	11.2 (2.6)	10.6 (2.8)
Clinical diagnosis			
ASD	9	32	382
Non‐ASD	10	53	216
No Dx information	2	2	20
IQ	97.3 (25.4)	94.0 (15.1)*	103.7 (17.7)
Verbal	96.2 (22.3)	94.55 (18.3)*	103.81 (17.7)
Nonverbal	102.8 (24.9)	94.50 (14.8)*	103.12 (17.7)
ADOS			
SA CSS	5.2 (3.0)	4.40 (2.9)*	5.22 (3.0)
RRB CSS	5.4 (3.4)	4.07 (3.2)*	5.58 (3.2)
Overall CSS	4.9 (3.1)	4.02 (2.8)*	5.10 (3.1)
SCQ Total Score	14.3 (10.2)	11.77 (9.5)*	16.07 (9.3)
SRS‐2			
Total T‐score	61.8 (16.9)	62.1 (15.8)*	68.7 (14.9)
SCI T‐score	62.2 (16.4)	62.2 (15.2)*	68.1 (14.6)
RRB T‐score	59.2 (16.8)*	60.7 (16.4)*	68.7 (15.3)

*Note:* Descriptives of racial groups are presented as mean (standard deviation). Asterisks denote a significant difference from the reference White group (*p* < 0.05).

Abbreviations: ASD, Autism Spectrum Disorder; SA CSS, Social Affect comparison score; RRB CSS, Restricted and Repetitive Behavior comparison score; IQ, Intelligence Quotient; Overall CSS, Overall comparison score; SCQ, Social Communication Questionnaire; SRS‐2, Social Responsiveness Scale, Second Edition; SCI, Social Communication and Interaction; RRB, Restricted Interests and Repetitive Behavior.

The Black/African American sample showed significantly lower scores (indicating fewer autistic features) than the reference White sample on the two caregiver‐report questionnaires (Tables S1–S3), and correlations between questionnaires and ADOS score were low (Tables S4 and S5). The Asian sample did not show significant differences from the reference White sample on any measure. Age showed no relationship with latent autistic traits (*r*(676) = −0.05, *p* = 0.18), and IQ showed a small negative relationship such that IQ decreased with increasing latent autistic trait levels, as may be expected (*r*(676) = −0.30, *p* < 0.001). In our sample, the sensitivity and specificity of ADOS classification for predicting clinical best estimate autism diagnosis were 0.894 and 0.817, respectively, with an accuracy rate of 0.863 (95% CI [0.836, 0.888]; Table S6); these results closely reflect the expected sensitivity (0.91) and specificity (0.84) for Module 3 (Lord et al. 2012; Carr 2013). Response frequencies for each ADOS item by racial group are provided (Tables S7 and S8).

### 3.2 Model Dimensionality

Goodness‐of‐fit indices demonstrated largely equivalent fits between the 1‐factor and unconstrained 3‐factor models; the constrained 3‐factor model demonstrated worse fit on most indices (Table 2). In order to avoid conducting underpowered multidimensional analyses given the smaller sample sizes of some racial subgroups, and to be consistent with previous research (Kalb et al. 2022), the unidimensional (1‐factor) model was selected for item analyses.

**TABLE 2:** Fit indices for confirmatory 1‐ and 3‐factor graded response models.

	M2	df	RMSEA	SRMSR	TLI	CFI
Unidimensional	1429.45	231	0.085	0.067	0.907	0.916
Confirmatory 3‐Factor Model (Allowing Covariance)	1298.87	228	0.081	0.066	0.916	0.925
Confirmatory 3‐Factor Model (Not allowing Covariance)	2056.60	231	0.104	0.206	0.859	0.872

*Note:* The 3‐factor model without covariance showed the poorest fit.

Abbreviations: CFI, Comparative Fit Index; df, degrees of freedom; RMSEA, Root Mean Square Error of Approximation; SRMSR, Standardized Root Mean Squared Residual; TLI, Tucker– Lewis Index.

### 3.3 Differential Item Functioning

**FIGURE 1:**
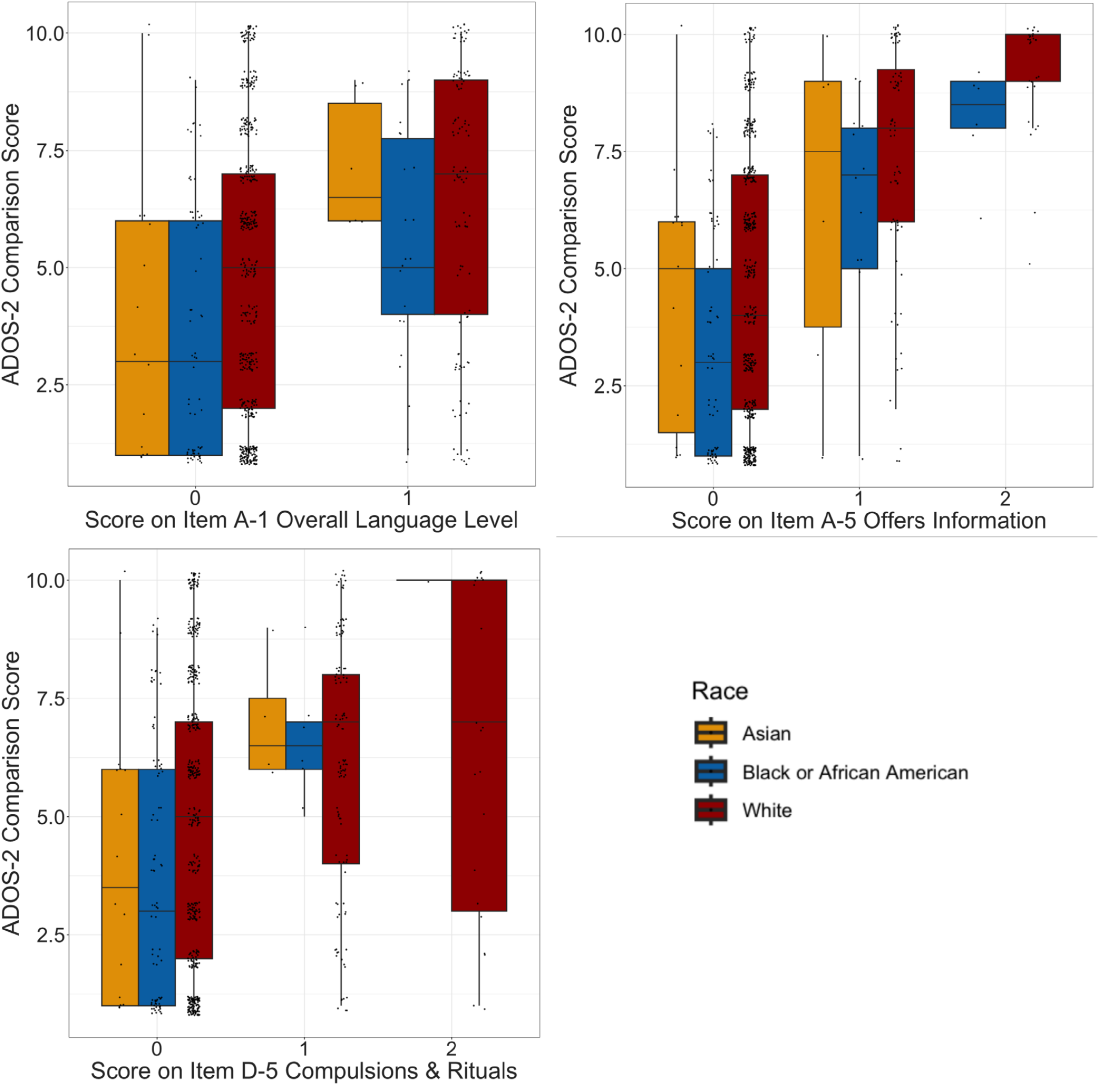
Box Plots of ADOS‐2 Comparison Score by Item Scores. Three items showed significant Differential Item Functioning: A1, A5, and D5. For each racial group, at each item score of 0, 1, or 2, the figures depict the boxplot (mean and distribution, with an overlaid datapoint for each participant) of the ADOS‐2 Comparison Score (CSS), a proxy for total score and level of autism. No Asian participants received scores of 2 on these three items (see Table S7), so no boxplot appears for an item score of 2. Few Black/African American participants received scores of 2 on items A1 and D5, so the mean but not interquartile range is depicted (see Table S7).

**FIGURE 2:**
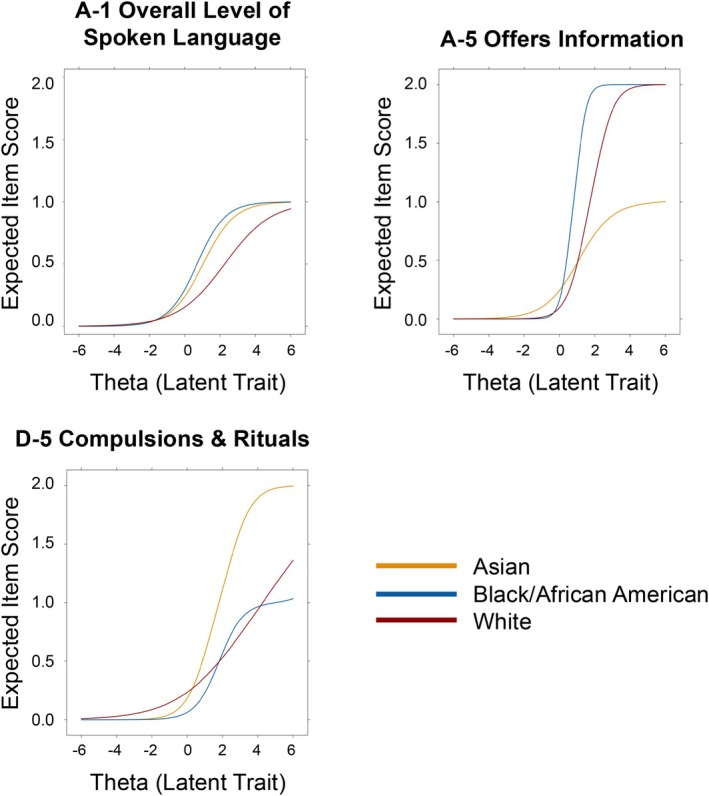
Item Characteristic Curves are depicted for the three items showing significant Differential Item Functioning. Theta reflects the latent trait of autism for this sample, standardized to a mean of 0 and standard deviation of 1. The expected score refers to the scores on the given ADOS item of 0, 1, or 2. The logistic function portrays the probability of a given score on the item, given the latent trait level. For item A5, Black/African American children were likely to receive a higher item score than White children, the reference group. This pattern held true at all levels of the latent trait (i.e., level of autism), called uniform DIF. In contrast, Asian children were likely to receive a lower item score than White children only at high levels of the latent trait (greater than approximately 1.0; nonuniform DIF).

Three items were consistently flagged for DIF (*p* < 0.05) in our baseline and final DIF detection models (Table 3): A1 (Overall Level of Non‐Echoed Spoken Language), A5 (Offers Information), and D5 (Compulsions or Rituals). The Black/African American and Asian groups showed distinct patterns compared to the reference White group (Figures 1 and 2) and will be discussed in turn. Of note, item A1 (Overall Level of Non‐Echoed Spoken Language) showed significant DIF both with and without excluding individuals who scored a 2 or 3.

**TABLE 3:** Items flagged for significant differential item functioning (DIF).

	χ2	*p*	a	*b*_1_	*b*_2_	DIF type	UIDS	SIDS	ESSD
A‐1 Overall Level of Non‐Echoed Spoken Language	10.92	0.027							
Asian			1.116	1.039	—	Nonuni.	0.106	0.105	0.625
Black/African American			1.244	0.700	—	Nonuni.	0.115	0.114	0.844
White			0.756	2.265	—				—
A‐5 Offers Information	22.98	0.001							
Asian			1.049	1.082	10.681	Nonuni.	0.148	0.040	0.140
Black/African American			3.775	0.464	1.129	Nonuni.	0.154	0.147	0.455
White			1.968	1.214	2.256				—
D‐5 Compulsions or Rituals	14.52	0.024							
Asian			1.547	1.045	2.561	Nonuni.	0.140	0.040	0.165
Black/African American			1.485	1.817	8.218	Uniform	0.142	−0.142	−1.777
White			0.565	2.399	6.166				—

*Note:* A chi‐squared statistic and *p*‐value were computed for each DIF item in the three‐group model; the White group was specified as the reference group. Item parameters including discrimination (a), difficulty of scoring a 1 (*b*_1_), and difficulty of scoring a 2 (*b*_2_) were computed for each item for each group in the final model; higher numbers reflect greater difficulty and better discrimination. The effect sizes of signed item difference in the sample (SIDS) and unsigned item difference in the sample (UIDS) are presented with units of item points, such that a UIDS value of 0.14 indicates that Black/African American participants average a 0.14‐point increase on the ADOS item. The expected standardized score difference (ESSD) expresses the SIDS in standard deviation units and can be interpreted as one would interpret Cohen's *d*.

Abbreviation: DIF, Differential Item Functioning.

#### 3.3.1 DIF For Black/African American Participants

The ADOS item A1 (Language Level) exhibited lower difficulty parameters for Black/African American children, meaning that, on average, clinicians were more likely to rate Black/African American children as having a lower language level than White children with the same overall level of latent autistic traits (autism latent trait, in measurement parlance; Table 3). A Black/African American child received a score 0.12 points higher (reflecting lower language level) than a White child with an equivalent latent level of autism, which corresponds to a large effect size (ESSD = 0.844) according to conventional Cohen's *d* interpretation guidelines. The item showed nonuniform DIF; the discrepancy was especially pronounced for children with higher levels of autistic traits.

For ADOS item A5 (Offers Information), the effect size was similar; Black/African American children received a score 0.46 points higher than similar White peers, and the effect was more pronounced at higher autistic trait levels (nonuniform DIF). The effect was also similar in magnitude and direction for ADOS item D‐5 (Compulsions or Rituals); however, zero Black/African American children received a score of 2 on this item, thereby obscuring the clear interpretation of difficulty (*b*_2_ parameter) and effect sizes (UIDS, SIDS, ESSD). Most DIF effect size measures are computed by transforming the expected score differences between the focal group and the reference group. In cases such as item D‐5 where far fewer focal group than reference group individuals receive high item scores, these statistics become heavily biased toward the reference group (Meade 2010).

#### 3.3.2 DIF For Asian Participants

ADOS item A1 (Language Level) was found to be less difficult for Asian children, meaning that, on average, clinicians were more likely to rate Asian children as having a lower language level than White children (Asian *b*_1_ = 1.12; White *b*_1_ = 0.76) with the same overall level of autistic traits (autism latent trait, in measurement parlance). An Asian child received a score 0.11 points higher (reflecting a lower language level) than a White child with an equivalent level of autism, which corresponds to a moderate effect size (ESSD = 0.63). The discrepancy was most pronounced at high levels of autistic latent trait (nonuniform DIF).

For ADOS item A5 (Offers Information), the pattern of results was similar. An Asian child received a score of 0.15 points higher (reflecting less frequently offering information about themselves in conversation) than a White child with an equivalent level of autism. The effect size was smaller due to cancellation caused by nonuniform DIF (ESSD = 0.14), meaning that the item was overrated for some participants but underrated for others, based on their ADOS total score (Figure 2). None of the 21 Asian children received the highest score of 2, inconsistent with the larger proportion of White children receiving a 2 (5% = 29/618; Figure 2).

Finally, for ADOS item D5 (Compulsions or Rituals), the effect was small and nonuniform. Clinicians were more likely to rate Asian children as exhibiting the behavior than White children with the same overall level of autistic traits, and this bias was particularly pronounced at higher levels of autism. The overall effect size was small, again due to cancellation (ESSD = 0.17; Table 3).

### 3.4 Test‐Level Effect of Differential Item Functioning

In terms of the impact of the three DIF items on the ADOS total score, none of the items showing DIF are “algorithm items” and therefore are not included in the ADOS score used to classify as autism, autism spectrum, or non‐spectrum. Thus, there is little or no impact of DIF on the algorithm score in this sample. Furthermore, the UETSDS effect size showed that if all 24 items were summed for a total score with a range of 0–48, the overall impact of the three DIF items would be quite small: the Black/African American children showed a 0.226 (SE 0.0056) point increase in total hypothetical ADOS score compared to White children in this sample, while the Asian children showed a 0.157 (SE = 0.0066) point increase compared to White children in this sample (Figure S1, Tables S9 and S10).

### 3.5 Power Analysis

The comparison of White and Black children was sufficiently powered to detect up to 10 DIF items (1‐*β* = 0.81), with a minimum DIF effect size of 1 and a significance level of 0.05. At the same DIF effect size, the comparison of White and Asian children did not have acceptable power (1‐*β* > 0.80) for detecting any number of DIF items.

## 4 Discussion

Using unidimensional item response theory graded response models, we identified 3 of 24 ADOS Module 3 items with statistically significant differential item functioning (DIF) by children's race: A1 (Overall Language Level), A5 (Offers Information), and D5 (Compulsions and Rituals). On these items, Black/African American and Asian children were usually more likely to be rated as showing autistic behaviors than White children with similar autism levels. For some items, the effect was more or less pronounced depending on autism levels. The impact of this racial bias on the item score was small, ranging from 0.08 to 0.15 ADOS points on the individual items. The impact on the overall test score was even smaller: on a scale of 0–48 points, the effect of racial bias was estimated at 0.23 total points for Black/African American children and 0.16 points for Asian children. Furthermore, none of the three items are “algorithm” items that contribute to the autism classification algorithm.

Two previous reports investigated differential item functioning on ADOS items between Black/African American and White participants. Both reports analyzed only algorithm items common across most modules and identified multiple items with significant DIF. However, only one item showed DIF in both studies: A4 (Stereotyped Language). We did not identify DIF in this or any of the previously reported DIF items. The three items that demonstrated DIF in our analysis were not studied in previous reports because they are not algorithm items. While it is somewhat surprising that so few results replicated between these three ADOS DIF analyses, these results may be explained by differences between the samples and other factors that can interact with racial DIF (sex DIF, ADOS version, clinician training, etc.). Both Kalb et al. (2022). and our study concluded that the overall impact of racial DIF was minimal. Harrison et al. (2017). employed a two‐factor model and found the impact of DIF was meaningful for the factor associated with restricted and repetitive behaviors and interests. Further analyses of all Module 3 items will be necessary to replicate our results.

Several possible mechanisms could underlie the item DIF identified in this and other reports. First, it is important to note that no biological mechanism has been posited whereby racial differences in autism presentation would be expected. However, cultural differences in social norms (e.g., norms around eye contact in Asian cultures) may influence the presentation of autism and what is considered different from the norm (and thus scored as “abnormal”). (Harrison et al. 2017) A potentially relevant variable is the cultural lens through which the clinician evaluates behavior. However, clinicians' race and cultural background were unavailable in the present dataset. One might argue that the rigorous process to attain reliability (agreement between clinicians on 80% of codes) should attenuate the impact of clinician background culture on scores; however, a racially or culturally homogeneous group of clinicians who agree on 80% of scores may simply be congruent in their bias. Furthermore, vestigial bias may exist in the 20% of codes on which clinicians might disagree or drift apart over time. Future studies should examine the effects of clinician demographic variables on ADOS scoring.

With regard to interpreting results on item A1, DIF was detected between scores of 0 (“Uses sentences in a largely correct fashion”) and 1 (“Speech [has] recurrent grammatical errors not associated with use of dialect”). Thus, Black/African‐American and Asian children were rated as speaking with a different number of grammatical errors than White children with equivalent autistic traits, especially at higher levels of the autism latent trait. The current sample lacks the crucial data on participant language (e.g., participant and examiner dialect, participant's exposure to multiple languages, co‐occurring developmental language disorders, etc.) required to provide an accurate explanation for the DIF observed on this item; future research involving linguists or speech‐language pathologists is warranted.

With regard to interpreting results on items A5 (Offers Information) and D5 (Compulsions and Rituals), Black/African‐American children were likely to be overscored (i.e., scored higher, as showing the autistic behavior) than White peers with equivalent levels of autism. The exception was that no Black/African‐American children received the highest score of 2 on D5 (Compulsions and Rituals). This pattern corroborates parent report on the RRB subscale of the SRS‐2, which suggested that Black/African‐American children in this sample show significantly less RRBIs than White children.

Asian children were also more likely to be overscored on these two items. The exception was that no Asian children received the highest score of 2 on A5 (Offers Information), meaning that Asian children tended to share appropriate personal information about their thoughts, feelings, or experiences with the examiner on several occasions (score of 0) or occasionally (score of 1). No clear cultural consideration explains why Asian children were more likely to be overscored, meaning rated as 1 rather than 0. With regard to overscoring on D5 (Compulsions and Rituals), clinicians were more likely to rate the child as showing this autistic behavior, but on average parents also rated Asian children in this sample as showing significantly fewer RRBIs than White children. We speculate that these overscoring discrepancies may stem from these behaviors being more salient to clinicians when evaluating Asian children, but further research is needed to better understand their origins.

Although we observed racial bias with very small effect sizes and no significant impact on ADOS autism classification, studying DIF in ADOS items still offers valuable information. First, the IRT discrimination parameter *a* highlights which items are discriminating well and for which groups, which is helpful given the challenge of diagnosing autism. For example, many innovative mobile diagnostic tools and devices are being developed to increase accessibility to autism diagnosis in rural areas (Antezana et al. 2017). These diagnostic tools often rely on a subset of autistic features to make a diagnosis, so it is important to know how well features rated in the ADOS discriminate between autistic and nonautistic individuals within each racial group. For example, in our analysis, A1 (Overall Language Level) and D5 (Compulsions and Rituals) showed better discrimination for Black/African American and Asian children than for White children, while A5 (Offers Information) showed worse discrimination for Asian children. In other words, Asian children with high levels of autism do not necessarily receive a high score on Offers Information, but they are likely to receive high scores on Language Level and Compulsions and Rituals; these items are better able to discriminate between autistic and nonautistic Asian children. As innovative efforts are made to decrease healthcare disparities, racial equity can be approached from a data‐driven perspective. Specifically, IRT and DIF analyses can be conducted on all outcome metrics (treated as items) of new tools, and those metrics with similar item parameters across racial groups (i.e., no significant DIF) can be prioritized. Similarly, it is important to know which items have a higher difficulty parameter (*b*_1_, *b*_2_), meaning that clinicians are less likely to rate the behavior as present in a particular racial group, so that clinicians can keep that potential bias in mind while scoring and compensate.

### 4.1 Limitations

DIF analysis requires large sample sizes in all groups, and the availability of only 21 Asian and 87 Black/African American participants led to lower confidence in our model selections and insufficient power for the Asian model. There may be other DIF effects that this sample of 735 participants was not sufficiently powered to detect. It should be noted that while the three items with significant DIF all included at least one empty cell (e.g., no “2” scores for the Asian subgroup on A5), the impact of empty cells did not overwhelm the analysis, as many other items included empty cells but did not have significant DIF. Second, since data were combined across 14 studies, there are some systematic sample differences due to ascertainment, the structure of each study's clinical team, and how the ADOS was administered. Furthermore, since data were collected over 15 years and thus used both ADOS and ADOS‐2, we combined these datasets to maximize our power to detect DIF. Some items changed wording, but we argue these differences are no more different than the discrepancies in constructs and codes between modules (Harrison et al. 2017). Finally, our analysis rests on 24 Module 3 items, not only the algorithm items. Non‐algorithm items comprise the majority of the ADOS and contribute substantially to overall clinical impression; this DIF analysis of all ADOS items provides added value to the ongoing conversation about DIF in the ADOS and disparities in the overall diagnostic process.

### 4.2 Conclusions

This paper provides the first analysis of racial bias in ADOS administrations for Asian children, as well as the only analysis to our knowledge that includes all ADOS items (instead of algorithm items only). Three items showed differential item functioning that may reflect racial bias around Black/African American and Asian children. However, the impact of these differences on overall ADOS scores was negligible, suggesting that observed item biases are unlikely to have a significant impact on diagnostic decision‐making. The present results suggest that bias in scoring for Black/African American and Asian children relative to White children on the ADOS Module 3 items may be rather modest. Nonetheless, because of the potential for significant consequences, we advocate for DIF analysis of other large ADOS data sets and further research into mechanisms that may underlie observed disparities in autism‐related health care.

## Funding

This work was supported by the National Institute of Mental Health (R42MH115539, R01MH125958, K23MH086111, R01MH084961, R21MH092615, R01MH133838, R21MH129777), National Institute of Child Health and Human Development (P50HD105354, 5P50HD103555), National Institute on Deafness and Other Communication Disorders (R01DC018289).

## Ethics Statement

De‐identified data were analyzed from 14 research studies conducted at the Center for Autism Research at the Children's Hospital of Philadelphia from 2009 to 2024 (IRB # 23‐021354).

## Consent

All participants provided informed consent.

## Conflicts of Interest

Yu, Wyman, Faulk, Fulop, Greenberg, Benecke, Steinbeck, Foy, Kim, Emory, Zampella, Yerys, Schultz, Parish‐Morris, Herrington, and Clements report no conflicts of interest. Storch reports receiving research funding to his institution from the Ream Foundation and International OCD Foundation. He was a consultant for Brainsway and Biohaven Pharmaceuticals in the past 12 months. He owns stock less than $5000 in NView (for distribution of the Y‐BOCS and CY‐BOCS). He receives book royalties from Elsevier, Wiley, Oxford, American Psychological Association, Guildford, Springer, Routledge, and Jessica Kingsley.

## Supporting information


**Data S1:** aur70155‐sup‐0001‐Supinfo.docx.

## Data Availability

De‐identified data are available from the authors upon reasonable request.

## Footnotes

^1^
The language here (e.g., “abnormal”) is quoted from the scoring guidance in the ADOS manual. The authors of this article endorse current recommendations to use neurodiversity affirming language in research and clinical practice (Botha et al. 2021).
