# Supplementary material for: An Examination of Racial Bias in Scoring the Autism Diagnostic Observation Schedule (ADOS) Module 3: An Item Response Theory Analysis
Source: Autism Res. 2025 Dec 23;19(2):e70155. doi: 10.1002/aur.70155 (PMC12948737; doi:10.1002/aur.70155)
Supplement: Supplementary file 1 — Data S1: aur70155‐sup‐0001‐Supinfo.docx. [file AUR-19-0-s001.pdf]

An Examination of Racial Bias in Scoring the Autism Diagnostic Observation Schedule (ADOS) Module 3

Supplement

Supplemental Methods

Figures

Figure S1. Test Characteristic Curves.

Tables

Table S1. Participant characteristics by autism diagnosis.
Table S2. Participant ethnicity.
Table S3. Participant ethnicity by race and autistic status.
Table S4. Autism measure correlations
Table S5. Autism measure correlations by autism diagnosis
Table S6. Confusion matrix of ADOS classification.
Table S7. ADOS item response frequences by race.
Table S8. ADOS Domain C and E item response frequences by race.
Table S9. IRT statistics of all items.
Table S10. Test-level effect of differential item functioning.

Supplemental Methods

The 14 studies that contributed data to this secondary analysis addressed a broad range of primary research questions, including case-control analysis of behavioral and neuroimaging features in autistic versus typically developing children, the application of computer vision methods to distinguish phenotypic markers of autism from those of other psychiatric conditions such as anxiety and depression, the evaluation of anxiety-related biomarkers in children with and without autism and co-occurring anxiety, and investigation of special interests and cognitive control in children with ADHD and/or on the spectrum. Thus, included children represent a range of autism, other psychiatric conditions, and typical development .

The Autism Diagnostic Observation Schedule (ADOS) is widely regarded as a "gold standard" source of information about a child's behavior and social skills during the autism diagnostic process[9,10]. During the ADOS administration, the child and clinician engage in semi-structured interactive play and communication tasks designed to elicit and probe language use, toy and object use, social skills, interests, and behaviors. Activities are tailored to the child's age and language level through modules; for example, Module 3 is most appropriate for verbally fluent children from 4-15 years old, and thus, is very widely used. Example tasks from Module 3 include assembling a puzzle, make-believe play with action figures, and a structured interview about bullying and emotions. There are 14 tasks completed in Module 3, which lasts 45-60 minutes.

ADOS tasks are designed to elicit certain behaviors relevant to autism, which the clinician scores following the administration. Module 3 includes 29 items (or behaviors) that fall into five domains: A) language and communication (e.g., echolalia, intonation of speech, elaborations on responses for the examiner's benefit, gestures); B) reciprocal social interaction (e.g., frequency of eye contact, facial expressions, integration of facial expression with eye contact, quality of rapport, frequency and quality of reciprocal conversation); C) imagination (e.g., creative play or comments); D) stereotyped behaviors and restricted interests (e.g., finger tensing, sensory interests in toys); and E) other behaviors (e.g., anxiety, tantrums).

Following the administration, the clinician rates or scores each specified behavior. The clinician assigns a score of 2 or 3 if the behavior is "definitely" or "markedly abnormal," a score of 1 if the behavior is "mildly abnormal or slightly unusual," and a score of 0 if there is "no evidence of abnormality", though a score of 0 "does not necessarily imply that the behavior is normal."[11] Each score includes a detailed description with examples to guide clinician ratings

and the training in scoring is rigorous. Training in ADOS administration and scoring is overseen by Western Psychological Services and includes a 2- to 4-day training, followed by review of videotaped ADOS administrations and resulting scores. A “reliable” administrator has achieved consistent administration fidelity and 80% scoring reliability[12].

Scores from a subset of items are summed to create the total score (“algorithm score”). The total score has cut-offs for classification of autism, autism spectrum, and non-spectrum[11]. Of the 29 items in Module 3, 14 items (called “algorithm items”) are included in the total score algorithm. The total algorithm scores from both the original ADOS and the ADOS-2 can be converted to a harmonized Comparison Score (CSS), which ranges from 1-10[17]. Scores 9-10 are classified as “autism,” scores 7-8 are classified as “autism spectrum,” and scores under 7 are classified as “non-spectrum”[11,17]. ADOS classification does not constitute a diagnosis, but the clinician integrates this information with multiple other sources such as a developmental history, caregiver interview, and cognitive testing[14].

Figure S1. Test Characteristic Curves.

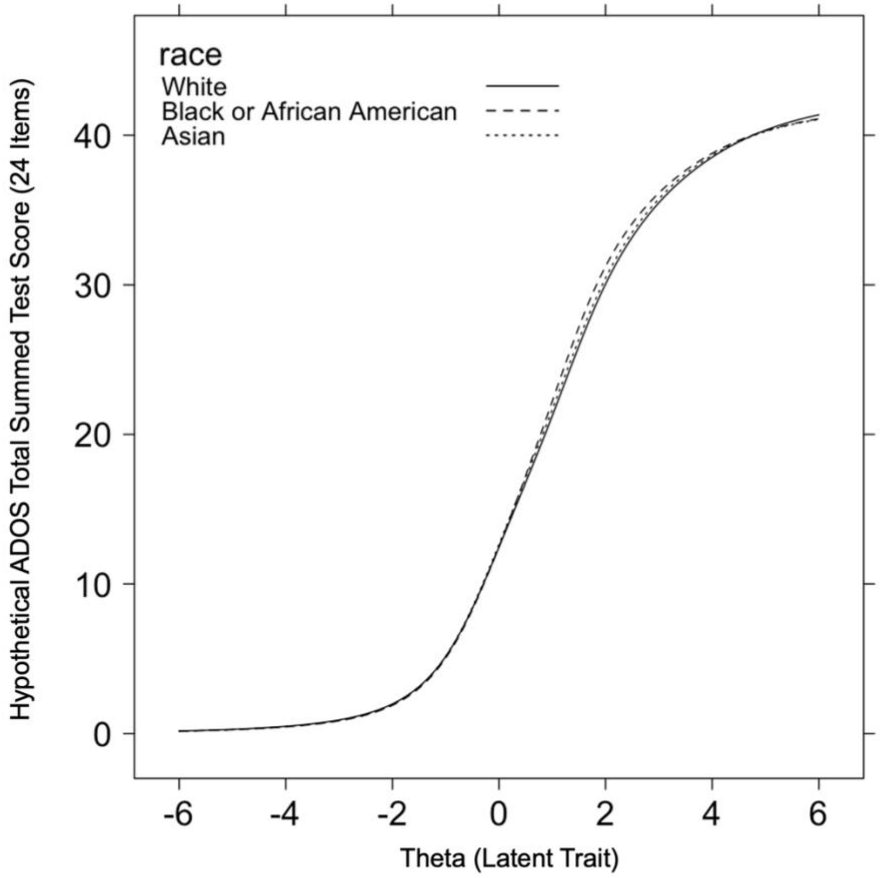

Test Characteristic Curves are depicted for the three racial groups. The similarity of the curves reflects the small effect of DIF on the overall test. Given the aggregated impact of all ADOS items, the overall ADOS test does not show clinically meaningful bias.

Table S1. Participant characteristics by autism diagnosis.

	Autistic			Non-autistic		
	**Asian**	**Black/ African American**	**White**	**Asian**	**Black/ African American**	**White**
N	9	32	382	10	53	216
Male	6	28	321	5	34	135
Female	3	4	61	5	19	81
Age (Years)	11.99 (1.73)	11.76 (2.21)	10.55 (2.68)	11.02 (2.85)	10.91 (2.73)	10.78 (2.96)
IQ	81.00 (27.63)	85.52 (15.19)*	100.85 (18.75)	112.00 (15.84)	98.63 (12.39)*	108.73 (14.77)
Verbal IQ	82.50 (24.35)	84.87 (21.65)*	100.92 (18.98)	108.10 (14.79)	99.88 (13.21)*	108.60 (14.49)*
Nonverbal IQ	90.75 (29.11)	88.14 (14.07)*	101.24 (18.75)	115.20 (17.51)	97.42 (13.64)*	106.39 (15.59)
ADOS						
SA CSS	7.00 (2.55)	6.78 (2.28)	6.75 (2.32)	2.90 (1.45)	2.98 (2.12)	2.75 (2.09)
RRB CSS	6.67 (2.65)	6.00 (2.85)*	7.07 (2.38)	3.60 (3.41)	3.01 (2.89)	3.19 (2.71)
Overall CSS	7.00 (1.94)	6.53 (1.98)	6.81 (2.35)	2.40 (1.84)	2.55 (2.02)	2.35 (1.87)
SCQ Total Score	21.89 (5.93)	17.69 (6.91)	20.08 (6.35)	7.60 (8.06)	8.00 (8.72)	9.52 (9.65)
SRS-2						
Total T-score	77.14 (13.92)	71.07 (9.85)	73.94 (10.85)	52.60 (11.04)	56.12 (15.31)	58.82 (16.54)
SCI T-score	76.71 (13.90)	70.80 (9.58)	73.09 (10.65)	53.50 (10.69)	56.41 (14.85)	58.56 (16.27)
RRB T-score	74.00 (12.78)	69.60 (11.11)*	74.10 (11.88)	49.80 (12.59)	54.53 (15.79)	58.79 (16.03)

Note. Descriptives of racial groups are presented as mean (standard deviation). Asterisks denote a significant difference from the reference White group ($p<0.05$).

Abbreviations: ASD, Autism Spectrum Disorder; SA CSS, Social Affect comparison score; RRB CSS, Restricted and Repetitive Behavior comparison score; IQ, Intelligence Quotient; Overall CSS, Overall comparison score; SCQ, Social Communication Questionnaire; SRS-2, Social Responsiveness Scale, Second Edition; SCI, Social Communication and Interaction; RRB, Restricted Interests and Repetitive Behavior.

Table S2. Participant ethnicity

	Hispanic/Latino	Not Hispanic/Latino	Not reported
Total	35 (4.8%)	661 (91.0%)	30 (4.1%)
Male	23 (4.2%)	498 (91.4%)	24 (4.4%)
Female	12 (6.6%)	163 (90.1%)	6 (3.3%)
Race			
Asian	0 (0%)	20 (95.2%)	1 (4.8%)
Black/African American	3 (3.4%)	79 (90.8%)	5 (5.7%)
White	32 (5.2%)	562 (90.9%)	24 (3.9%)
Clinical Diagnosis			
ASD	19 (4.5%)	386 (91.3%)	18 (4.3%)
Non-ASD	16 (5.7%)	251 (90.0%)	12 (4.3%)

Note. Descriptives are presented in N (%). Participants without autism diagnosis information (n=24) are not included in the clinical diagnosis columns. Percentages may not add up to 100.0% due to rounding.

Table S3. Participant ethnicity by race and autistic diagnostic status

	Autistic			Non autistic		
	Asian	Black	White	Asian	Black	White
Hispanic or Latino	8	3	16	10	0	16
Not Hispanic or Latino	0	28	350	0	49	192
Not reported	1	1	16	0	4	8

Note. Participants without autism diagnosis information (n=24) are not included in this table.

Table S4. Autism measure correlations

		Correlation	
		**Pearson**	**Spearman**
**ADOS-2 Overall CSS**	**SCQ Total Score**	0.476*	0.457*
**ADOS-2 Overall CSS**	**SRS-2 T-Score**	0.428*	0.422*
**SCQ Total Score**	**SRS-2 T-Score**	0.785*	0.750*

Note: Asterisks denote significant correlations between scores.
Abbreviations: Overall CSS, Overall comparison score; SCQ, Social Communication Questionnaire; SRS-2, Social Responsiveness Scale, Second Edition.

Table S5. Autism measure correlations by autism diagnosis

		Autistic		Non-Autistic	
		**Pearson**	**Spearman**	**Pearson**	**Spearman**
**ADOS-2 Overall CSS**	**SCQ Total Score**	0.046	0.049	0.214*	0.329*
**ADOS-2 Overall CSS**	**SRS-2 T-Score**	0.072	0.088	0.247*	0.347*
**SCQ Total Score**	**SRS-2 T-Score**	0.518*	0.500*	0.854*	0.844*

Note: Asterisks denote significant correlations between scores.
Abbreviations: Overall CSS, Overall comparison score; SCQ, Social Communication Questionnaire; SRS-2, Social Responsiveness Scale, Second Edition.

Table S6. Confusion matrix of ADOS classification and clinician-determined autism diagnosis.

		ASD DSM-5 Diagnosis	
		**ASD**	**No ASD**
ADOS Classification			
	Autism/Autism Spectrum	378	51
	Non-Spectrum	45	228

Note. Participants without autism diagnosis information (n=24) are not included in this table. Comparison of ADOS-2 classification and ASD diagnosis as confirmed by an expert clinician using DSM-5 diagnostic criteria. All ADOS classifications were determined using the ADOS-2 scoring cutoffs: algorithm total scores range from 0-28; scores 9-10 are classified as “autism,” scores 7-8 are classified as “autism spectrum,” and scores under 7 are classified as “non-spectrum” (Lord et al., 2012). The algorithm total score is also converted to a Comparison Scores (CSS) based on chronological age, and CSS’s range from 1-10 (Hus et al., 2014).
Abbreviations: ADOS, Autism Diagnostic Observation Schedule; ASD, Autism Spectrum Disorder; DSM-5, Diagnostic and Statistical Manual 5th Edition; CSS, comparison score.

Table S7. ADOS item response frequencies by race.

	Asian			Black/African American			White		
**Score on ADOS item**	**0**	**1**	**2**	**0**	**1**	**2**	**0**	**1**	**2**
A-1 Overall Level of Non-Echoed Spoken Language	15	6	*NA*	65	22	*NA*	509	109	*NA*
A-2 Speech Abnormalities Associated with Autism (Intonation/Volume/Rhythm/Rate)	9	7	5	45	33	9	216	237	165
A-3 Immediate Echolalia	17	3	1	82	5	0	584	29	5
A-4 Stereotyped/Idiosyncratic Use of Words or Phrases	10	7	4	55	24	8	286	239	93
A-5 Offers Information	15	6	0	69	12	6	509	80	29
A-6 Asks for Information	10	7	4	31	26	30	205	227	186
A-7 Reporting of Events	9	11	1	55	27	5	363	202	53
A-8 Conversation	11	6	4	45	31	11	251	269	98
A-9 Descriptive, Conventional, Instrumental or Informational Gestures	13	7	1	54	32	1	398	191	29
B-1 Unusual Eye Contact	10	*NA*	11	47	*NA*	40	290	*NA*	328
B-2 Facial Expressions Directed to Examiner	10	11	0	44	34	9	272	297	49
B-3 Language Production and Linked Nonverbal Communication	19	2	0	72	14	1	531	85	2
B-4 Shared Enjoyment in Interaction	12	6	3	53	25	9	327	186	105
B-5 Comments on Others' Emotions/Empathy	9	9	3	36	29	22	196	219	203
B-6 Insight Into Typical Social Situations and Relationships	2	8	11	34	25	28	153	198	267
B-7 Quality of Social Overtures	9	9	3	45	41	1	237	308	73
B-9 Quality of Social Response	4	14	3	31	53	3	172	382	64
B-10 Amount of Reciprocal Social Communication	13	5	3	64	18	5	356	196	66
B-11 Overall Quality of Rapport	9	9	3	54	26	7	273	253	92
D-1 Unusual Sensory Interest In Play Material/Person	13	6	2	72	10	5	419	123	76

D-2 Hand and Finger and Other Complex Mannerisms	17	0	4	79	7	1	499	62	57
D-3 Self-Injurious Behavior	21	0	0	87	0	0	614	4	0
D-4 Excessive Interest in or References to Unusual or Highly Specific Topics or Objects or Repetitive Behaviors	13	5	3	57	23	7	346	170	102
D-5 Compulsions or Rituals	16	4	1	81	6	0	483	114	21

Abbreviations: ADOS, Autism Diagnostic Observation Schedule
Note. Scores such as 3, 7, 8, or 9 were converted to 0 or 2 in accord with ADOS conventions (*i.e.,* 3s to 2s, and 7s, 8s, and 9s, to 0).

Table S8. ADOS Domain C and E item response frequences by race.

	**Asian**			**Black/African American**			**White**		
**Score on ADOS item**	**0**	**1**	**2**	**0**	**1**	**2**	**0**	**1**	**2**
C-1 Imagination/Creativity	11	8	2	50	32	5	290	247	81
E-1 Overactivity/Agitation	16	3	2	67	13	7	403	165	50
E-2 Tantrums, aggression, negative or disruptive behavior	21	0	0	85	1	1	575	36	7
E-3 Anxiety	20	0	1	79	8	0	478	124	16

Note. Chi-square tests of independence were conducted to examine whether score distributions differed by race for each ADOS-2 item within the C and E domains. Significant group difference was found for item E-3 (Anxiety; $\chi^2(4) = 14.03$, $p = 0.007$), but not for items C-1 (Imagination/Creativity; $\chi^2(4) = 5.56$, $p = 0.234$), E-1 (Overactivity/Agitation; $\chi^2(4) = 7.13$, $p = 0.129$), and E-2 (Negative/Aggressive behavior) ($\chi^2(4) = 4.88$, $p = 0.3$).

Table S9. IRT statistics of anchor items

	a	b1	b2
A-1 Overall Level of Non-echoed Spoken Language			
A-2 Speech Abnormalities Associated with Autism (Intonation/Volume/Rhythm/Rate)	1.708	-0.500	0.925
A-3 Immediate Echolalia	1.483	2.381	3.886
A-4 Stereotyped/Idiosyncratic Use of Words or Phrases	1.279	-0.116	1.710
A-5 Offers Information	1.968	1.214	2.256
A-6 Asks for Information	0.502	-1.452	1.704
A-7 Reporting of Events	1.472	0.282	2.122
A-8 Conversation	2.850	-0.270	1.127
A-9 Descriptive, Conventional, Instrumental or Informational Gestures	0.969	0.660	3.594
B-1 Unusual Eye Contact	1.559	-0.123	-
B-2 Facial Expressions Directed to Examiner	1.695	-0.218	1.983
B-3 Language Production and Linked Nonverbal Communication	0.364	4.994	15.200
B-4 Shared Enjoyment in Interaction	1.813	0.080	1.312
B-5 Comments on Others' Emotions/Empathy	1.091	-0.846	0.830
B-6 Insight Into Typical Social Situations and Relationships	1.643	-0.974	0.241
B-7 Quality of Social Overtures	2.526	-0.337	1.462
B-9 Quality of Social Response	2.741	-0.711	1.486
B-10 Amount of Reciprocal Social Communication	2.418	0.242	1.500
B-11 Overall Quality of Rapport	2.393	-0.152	1.267
D-1 Unusual Sensory Interest In Play Material/Person	0.887	1.015	2.557
D-2 Hand and Finger and Other Complex Mannerisms	0.740	2.205	3.432
D-3 Self-Injurious Behavior	-0.089	-58.327	-
D-4 Excessive Interest in or References to Unusual or Highly Specific Topics or Objects or Repetitive Behaviors	0.865	0.324	2.147

Table S10. Test-level effect of differential item functioning

	STDS	UTDS	UETSDS	ETSSD
White and Black/African American	0.119	0.411	0.227	0.019 (0.0038)
White and Asian	0.185	0.394	0.191	0.023 (0.0039)

Note. Standard Errors of ETSSDs were generated by bootstrapping and are indicated in parentheses.

Abbreviations: ETSSD, Expected Test Standardized Score Difference; STDS, Signed Test Difference; UETSDS, Unsigned Expected Test Score Differences in the Sample; UTDS, unsigned test difference
